# Quantifying the effectiveness of rehabilitation for post‐radical prostatectomy urinary incontinence: Novel pelvic floor function assessment using M‐mode ultrasonography

**DOI:** 10.1002/iju5.12476

**Published:** 2022-05-29

**Authors:** Yukimasa Ide, Nobuki Kikuchi, Tetsuya Fukumoto, Hajime Takeda, Takafumi Okura

**Affiliations:** ^1^ Department of Physical Therapy Yawatahama City General Hospital Ehime Japan; ^2^ Department of Urology Ehime University, Graduate School of Medicine Ehime Japan; ^3^ Department of Urology Yawatahama City General Hospital Ehime Japan; ^4^ Department of Internal Medicine Yawatahama City General Hospital Ehime Japan

**Keywords:** bladder base elevation time, pelvic floor muscle training, physical therapists, post‐radical prostatectomy urinary incontinence, urinary bladder M‐mode ultrasonography

## Abstract

**Introduction:**

This study aimed to develop a method for evaluating pelvic floor function using M‐mode ultrasound imaging and quantify the effectiveness of urinary incontinence rehabilitation.

**Case presentation:**

Eight participants aged 66–76 years, with urinary incontinence following radical prostatectomy, underwent pelvic floor muscle training. Using M‐mode ultrasound, we compared bladder base elevation time, length, and speed during pelvic floor muscle contraction. The results showed that four patients recovered urinary continence. Four patients displayed a 38.4%–80.1% reduction in urinary incontinence volume. Bladder elevation time was significantly reduced in all patients. Moreover, elevation speed increased significantly. Bladder base elevation time was 0.1 s in all patients in the acquired urinary continence group.

**Conclusions:**

Reducing bladder base elevation time to <0.2 s might be essential to achieve urinary continence. An elevation time of ≥0.3 s indicated significant pelvic floor muscle dysfunction.

**Abbreviations & Acronyms**
BBE: Bladder base elevation
HoLEP: Holmium laser enucleation of the prostate
PT: Physical therapist
PFM: Pelvic floor muscles
PFMT: Pelvic floor muscle training
RP: Radical prostatectomy
UC: Urinary continence
UCA: Urinary continence acquired
UI: Urinary incontinence
UII: Urinary incontinence improved

**Keynote message**

Using M‐mode ultrasonography enabled an easy evaluation of the bladder base in terms of elevation time, length, and speed during PFM contraction. Reducing the bladder base elevation time to <0.2 s was essential to recover UC. An elevation time ≥0.3 s indicated significant PFM dysfunction.

## Introduction

Generally, RP is highly effective in prostate cancer, with 97% of the patients surviving for at least 5 years following surgery.^1^ However, RP may result in some complications, including UI.^2^, ^3^

A multidisciplinary treatment approach determined by urologists, physiotherapists, and other healthcare professionals is the therapy standard for post‐RP UI. In men, PFMT is recognized as the physiotherapeutic modality for post‐RP UI. Moreover, the European Association of Urology recommends this procedure in patients with post‐RP UI.^4^ Recently, we have reported on M‐mode ultrasonography‐aided interventions performed by PT as a treatment of post‐RP UI.^5^ Via rectifying incorrect conduct of exercises, UI that persisted >2 years following RP could have been reduced by 42%–100%. Herein, we report a simple evaluation method for pelvic floor function, using M‐mode ultrasonography, and our attempt to quantify the effectiveness of UI rehabilitation.

## Case presentation

From September 2020 to March 2021, PT initiated PFMT in eight patients with post‐RP UI, aged 66–76 years (mean, 72.3 years). The Ethical Committee of the Yawatahama City General Hospital approved this study (IRB number: 20210325–002). All patients provided written informed consent prior to enrollment. Out of these eight patients, four were recruited immediately post‐surgery and four were 3 months to 6 years post‐surgery. Moreover, RP was performed through laparotomy in four patients, robotic surgery in two, and laparoscopic surgery in two. In all cases, no nerve‐sparing technique was used.

Following PFMT with guidance by a PT, the bladder base was visualized by transabdominal ultrasound tomography, with scans obtained in the sagittal imaging plane. In addition, we evaluated BBE time, length, and speed during PFM contraction using the M‐mode system.

The parameters obtained upon the diagnosis of UC (Pad‐free) were compared with values before intervention. In patients without UC, the current (end of May 2021) parameters were compared to those before intervention.

**Fig. 1:**
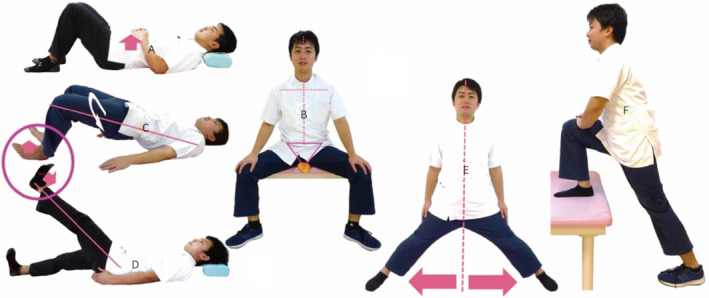
Methods of pelvic floor muscle training. (a) Abdominal breathing exercise and diaphragm training. (b) Pelvic floor muscle training: The exercise is performed to tighten the urethral sphincter and anal sphincter in the sitting position, a rolled towel is laid on the pelvic floor, both hip joints are abducted 45°, and the trunk is held in the intermediate position. (c) Strength training around the hip joint: hip external rotation and ankle dorsiflexion are added to the bridge exercise. (d) Hip‐strengthening exercise: ankle dorsiflexion is added to straight leg raising exercise. (e) Standing exercise: in addition to bilateral hip abduction, while one side shows hip joint flexing, the other side denotes hip joint extending. (f) Raise one foot on the table: with one foot raised on the table, applying a load that tilts the right hip bone backward and the left hip bone forward, with respect to the sacrum, is assumed as climbing a step or climbing a mountain.

Furthermore, PFMT involved a method to train the PFM, trunk, and hip joints in a coordinated process (Fig. 1). Instructed by a PT, PFMT sessions were continued at a frequency of 2–5 times/week for 2 weeks after the first intervention, followed by one session/month.

**Fig. 2:**
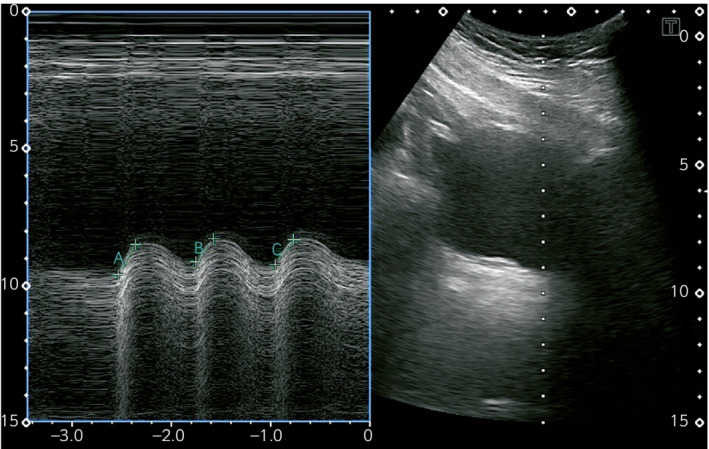
M‐mode ultrasound imaging. The bladder is displayed in the median sagittal imaging plane. The M‐mode cursor is set to the bladder base, which moves most at PFM contraction. Bladder base elevation time, length, and speed during PFM contraction are measured by using M‐mode ultrasound system. Bladder base elevation is measured at the highest point from the start point of PFM contraction.

Ultrasound evaluation was performed using Aplio 300 TUS‐A 300 (Canon Medical Systems Corporation, Otawara, Japan). Moreover, we used a 3.5 MHz convex‐type probe, with the patient placed in the supine position and the hip and knee joints slightly flexed. To detect the bladder base, urine was stored in the bladder for at least 60 min. The bladder base was visualized in the mid‐sagittal imaging plane in B‐mode, and the M‐mode cursor was set at the part with the largest range of motion during PFM contraction. We recorded the waveform (movement) of the bladder base during PFM contraction (Fig. 2) and analyzed it using the ultrasound device. Additionally, BBE was measured at the highest point from the start point of PFM contraction. The waveform of BBE was measured thrice, and the average value was used. Considering the absence of normal values of these parameters, BBE was evaluated by the same method in five healthy men in their 20s and in four patients with post‐HoLEP UCA (aged 75.2 ± 4 years) following PFMT, after obtaining their informed consent.

All analyses were performed using IBM SPSS 26.0 software (IBM, Armonk, NY, USA). The values are expressed as mean ± standard deviation. We performed the Wilcoxon signed rank test to compare the variables before and after PFMT. A *p*‐value <0.05 was considered statistically significant.

**Fig. 3:**
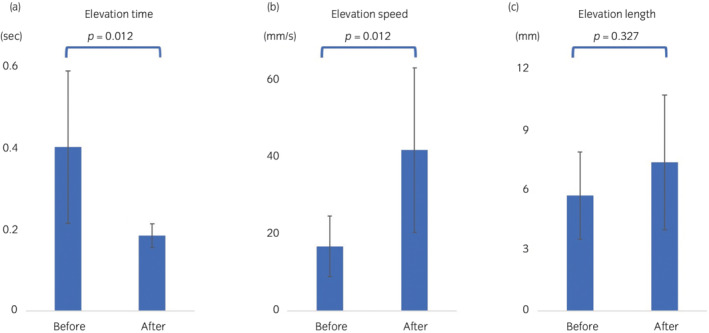
Bladder base elevation time, length, and speed before and after pelvic floor muscle training. (a) The bladder base elevation time significantly was reduced from 0.40 ± 0.18 s to 0.18 ± 0.02 s (*P* = 0.012). (b) The elevation speed significantly increases from 16.80 ± 7.87 mm/s to 41.87 ± 21.37 mm/s (*P* = 0.012). (c) The length of elevation tends to increase from 5.72 ± 2.17 mm to 7.36 ± 3.35 mm. However, the change is insignificant (*P* = 0.327) following PFMT.

Regarding results in all patients, although the BBE time was significantly reduced from 0.40 ± 0.18 s to 0.18 ± 0.02 s (*P* = 0.012) (Fig. 3a), the elevation speed significantly increased from 16.80 ± 7.87 mm/s to 41.87 ± 21.37 mm/s (*P* = 0.012) (Fig. 3b). The length of elevation tended to increase from 5.72 ± 2.17 mm to 7.36 ± 3.35 mm (Fig. 3c). However, the difference was insignificant (*p* = 0.327). Improved UI was observed in all cases, and four of the eight patients (Pad‐free) restored UC (Table 1A). Moreover, four patients had persistent UI. In patients with UII, the UI volume/day was reduced from 38.4% to 80.1% (Table 1B). In healthy men in their 20s, the BBE time, length, and speed were 0.18 ± 0.01 s, 7.36 ± 3.47 mm, and 40.42 ± 19.05 mm/s, respectively. In patients with post‐HoLEP UCA, the BBE time, length, and speed were 0.16 ± 0.03 s, 7.45 ± 5.23 mm, and 44.62 ± 29.5 mm/s, respectively.

**Table 1A:** Comparison of variables before and after the continuation of pelvic floor muscle training in the acquired urinary continence group

Case	Pre/Post	UI^*^ (g)	Time (s)	Length (mm)	Speed (mm/s)	Reduction ratio (%)
1	Pre	1845	0.2	5.5	28	100
	Post	0	0.183	5	28.1	
2	Pre	888	0.355	2.9	8.9	100
	Post	0	0.161	10.2	65.4	
3	Pre	500	0.25	4.8	19.2	100
	Post	0	0.175	4	22.9	
4	Pre	10	0.6	5	9.6	100
	Post	0	0.17	3.6	21.5	
Mean	Pre	810.7	0.351	4.55	16.42	100
	Post	0	0.172	5.7	34.47	

^*^ UI, urinary incontinence.

**Table 1B:** Comparison of variables before and after the continuation of pelvic floor muscle training in the urinary incontinence group

Case	Pre‐Post	UI^*^ (g)	Time (s)	Length (mm)	Speed (mm/s)	Reduction ratio (%)
1	Pre	978	0.503	5.7	11.6	61.5
	Post	377	0.209	4.7	24.8	
2	Pre	528	0.236	6.7	28.9	38.4
	Post	325	0.142	10.9	76.4	
3	Pre	402	0.361	4.8	13.5	80.1
	Post	80	0.231	8.9	40.1	
4	Pre	175	0.725	10.4	14.7	45.7
	Post	95	0.211	11.6	55.8	
Mean	Pre	520.7	0.456	6.9	17.17	56.4
	Post	219.2	0.198	9.02	49.27	

^*^ UI, urinary incontinence.

In the UCA group, all cases were within the 0.1 s range in elevation time. In contrast, three patients in the UII group had elevation time ≥0.2 s.

The BBE speed ≥20 mm/s was recorded in two of the eight patients before PFMT and in all patients following it.

## Discussion

In the UCA group, BBE time was substantially reduced, and all cases were within the 0.1 s range. Moreover, BBE time in all healthy men and in patients with post‐HoLEP UCA was also within this 0.1 s range. Therefore, BBE time <0.2 s was considered an index for restoring UC, by continuing PFMT, in patients with UI. In addition, BBE time did not record an average ≥0.3 s following the appropriate continuation of PFMT in all participants. Therefore, an average ≥0.3 s was considered an indicator of significant PFM dysfunction.

In all participants, BBE length did not significantly improve following PFMT. Miller et al. reported that the median vesical neck position in older women with UI was significantly dorsocaudal relative to its usual position among younger women.^6^ However, there is no stable reference landmark for reliable measurements of bladder base displacements during voluntary PFM contractions.^7^, ^8^ The length of BBE during PFM contraction may have been affected by PFM loosening. Hence, the demonstration of improvement in length of BBE following PFMT was difficult.

The BBE speed <20 mm/s was recorded in six of the eight patients before PFMT. Therefore, a BBE speed <20 mm/s might be associated with a high risk of UI.

## Conclusion

Pelvic floor function could be easily assessed by M‐mode ultrasonography. In addition, BBE time and speed were indicative of pelvic floor function and related to UI. Regardless of the duration of postoperative UI and age, improvement in UI or acquired UC could be expected. A BBE speed <20 mm/s might be associated with a high risk of UI. Reducing BBE time to <0.2 s was important to recover UC and ≥0.3 s was considered an indicator of significant PFM dysfunction.

## AUTHOR CONTRIBUTIONS

Yukimasa Ide: Conceptualization; data curation; investigation; methodology; project administration; writing – original draft. Nobuki Kikuchi: Validation; writing – review and editing. Tetsuya Fukumoto: Methodology; project administration; writing – review and editing. Hajime Takeda: Conceptualization; formal analysis; methodology; project administration; supervision; validation; writing – review and editing. Takafumi Okura: Formal analysis; project administration; resources; supervision; visualization; writing – review and editing.

## Conflicts of interest

The authors declare no conflict of interest.

## Approval of the research protocol by an Institutional Reviewer Board

The protocol for this research project has been approved by a suitably constituted Ethics Committee of the institution and it conforms to the provisions of the Declaration of Helsinki. The Committee of the Yawatahama City General Hospital, Approval number: 20210325–002.

## Informed consent

All informed consent was obtained from the participants.

## Registry and the Registration No. of the study/trial

N/A.
